# Hijacking microglial glutathione by inorganic arsenic impels bystander death of immature neurons through extracellular cystine/glutamate imbalance

**DOI:** 10.1038/srep30601

**Published:** 2016-08-01

**Authors:** Vikas Singh, Ruchi Gera, Rajesh Kushwaha, Anuj Kumar Sharma, Satyakam Patnaik, Debabrata Ghosh

**Affiliations:** 1 Immunotoxicology Laboratory, Food Drug and Chemical Toxicology Group and Nanotherapeutics & Nanomaterial Toxicology Group, CSIR-Indian Institute of Toxicology Research, Lucknow-226001, Uttar Pradesh, India; 2 Academy of Scientific and Innovative Research (AcSIR), CSIR-IITR Campus, Lucknow-226001, Uttar Pradesh, India; 3 System Toxicology Group, CSIR-Indian Institute of Toxicology Research, Lucknow-226001, Uttar Pradesh, India; 4 Water Analysis Laboratory, Nanotherapeutics and Nanomaterial Toxicology Group, CSIR-Indian Institute of Toxicology Research, Lucknow-226001, Uttar Pradesh, India

## Abstract

Arsenic-induced altered microglial activity leads to neuronal death, but the causative mechanism remains unclear. The present study showed, arsenic-exposed (10 μM) microglial (N9) culture supernatant induced bystander death of neuro-2a (N2a), which was further validated with primary microglia and immature neuronal cultures. Results indicated that arsenic-induced GSH synthesis by N9 unfavorably modified the extracellular milieu for N2a by lowering cystine and increasing glutamate concentration. Similar result was observed in N9-N2a co-culture. Co-exposure of arsenic and 250 μM glutamate, less than the level (265 μM) detected in arsenic-exposed N9 culture supernatant, compromised N2a viability which was rescued by cystine supplementation. Therefore, microglia executes bystander N2a death by competitive inhibition of system Xc^-^ (xCT) through extracellular cystine/glutamate imbalance. We confirmed the role of xCT in mediating bystander N2a death by siRNA inhibition studies. *Ex-vivo* primary microglia culture supernatant from gestationally exposed mice measured to contain lower cystine and higher glutamate compared to control and N-acetyl cysteine co-treated group. Immunofluorescence staining of brain cryosections from treated group showed more dead immature neurons with no such effect on microglia. Collectively, we showed, in presence of arsenic microglia alters cystine/glutamate balance through xCT in extracellular milieu leading to bystander death of immature neurons.

Microglia plays a dual role in neuroprotection as well as neurodegeneration depending on their activation status and incoming environmental stimuli^1^. Resting microglia behave friendly by clearing cellular debris, secreting neurotrophic factors and by recruiting immune cells for maintaining healthy central nervous system (CNS)^2^. In contrary, activated microglia adversely affect the brain environment by releasing toxic factors like, ROS, NO, IL-1β, and TNF-α, thereby inducing different neurodegenerative disorders^3^. Along with endogenous factors, various environmental toxicants including arsenic alter microglial function, which in turn initiate an inflammatory response in CNS^1^,^4^.

Arsenic became globally infamous for its toxic effect in every sphere of life despite its traditional use in Chinese and homeopathic medicine as well as efficient utilization in the treatment of different cancer^5^,^6^. Early life arsenic exposure causes an array of toxicity including neurotoxicity gaining global attention rapidly^7^. Although arsenic has been predicted to prime microglial activity eventually leading to neurodegenerative diseases like Alzheimer’s^8^ the underlying mechanism remains unexplored.

Arsenic can easily pass through blood-placenta^9^ as well as blood-brain barrier^10^ thereby risking the fetal growth and neuronal health in exposed adult^11^. The metalloid exerts its effect either by inhibiting enzyme function or by inducing oxidative stress^12^,^13^. The primary counteractive measure to oxidative stress is cellular GSH^14^,^15^. Depletion of neuronal and glial GSH can compromise neuronal viability leading to neurodegenerative disease symptoms^14^,^15^. GSH synthesis is dependent on the availability of cystine in the extracellular environment of developing brain. Immature neurons can exclusively uptake cystine through xCT and in return release glutamate in extracellular environment^15^,^16^. The exact role of xCT in neuroprotection or neurodegeneration is context dependent. Higher expression of xCT in the proximity of dentate gyrus and subventricular region has been found to meet the higher requirement of GSH by proliferating neuronal progenitor cells whereas, extracellular glutamate release can cause excitotoxicity in differentiated neurons^15^,^17^. Although, xCT^−/−^ knockout mice grow normally^18^ showing some atrophy^15^, but the effects of the presence of electrophilic agents such as arsenic remain elusive. Cortical neurons, especially developing/immature cells are more susceptible to cystine deprivation than glial cells^19^,^20^. Therefore, an abundance of cystine in the brain milieu is crucial for the developing brain to avoid cognitive dysfunction^21^,^22^,^23^. Cystine can be made unavailable to immature neurons by increased microglial intake for synthesizing GSH as well as by competitive inhibition of xCT by high extracellular glutamate level during stress^20^. Inorganic arsenic depletes cellular GSH by direct binding and efflux through MRP1^24^,^25^, thereby altering the cellular defense system. Arsenic-induced changes in cellular GSH level may alter extracellular cystine/glutamate balance which can compromise the viability of immature neurons owing to its higher susceptibility to low cystine/high glutamate level.

In the present study, we tested the hypothesis that arsenic-induced synthesis of microglial GSH alters extracellular cystine/glutamate balance leading to a bystander neuronal death. We investigated neuronal death induced by microglial culture supernatant in the presence of sodium arsenite (SA). We have checked the level of cellular ROS and GSH as well as NO, TNF-α, IL-6, glutamate, and cystine concentration in the microglial culture supernatant to find out the probable factor responsible for bystander neuronal death. Expression of xCT and MRP1 were also analyzed. *In-vitro* alterations of cystine/glutamate level and bystander death of immature neurons have been validated *in-vivo* by gestational exposure to arsenic with N-acetyl cysteine (NAC) supplementation. Cystine/glutamate concentrations measured in the *ex-vivo* culture supernatant of primary microglia isolated from gestationally treated pups. The death of developing neuron and microglia were detected by co-immunofluorescence labeling of brain cryosection with TUNEL and doublecortin (DCX) as well as TUNEL and Iba1. Here, we tried to delineate the role of microglia in inducing bystander death of immature neurons in the presence of arsenic.

## Results

### Sodium arsenite (SA)-exposed microglial culture supernatant induced bystander death of immature neurons

**Figure 1:**
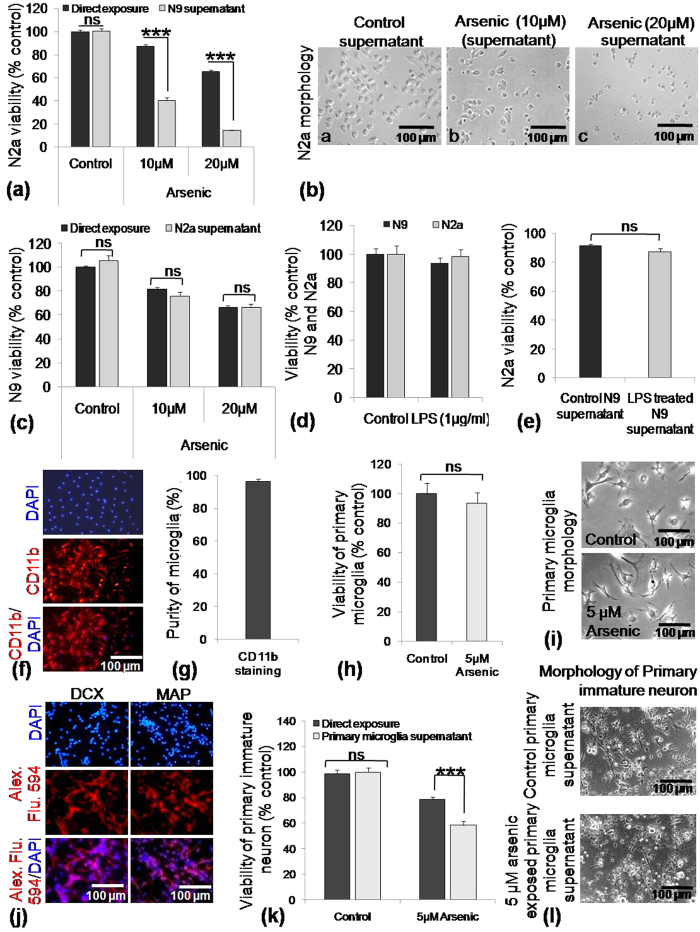
Effect of direct and indirect sodium-arsenite (SA) exposure on microglia and immature neuron culture. (**a**) N2a cells were either directly or indirectly (N9 culture supernatant grown in presence of arsenic for 24 h) exposed to 10 and 20 μM of SA for 24 h in 96 well culture plate and viability evaluated by MTT assay. N2a viability recorded to be 84% and 68% following 10 and 20 μM direct SA exposure, whereas in indirect exposed group N2a viability reduced to 40.1% and 14.6% respectively. (**b**) Representative photomicrograph of N2a culture 24 h followed by indirect SA exposure. (**c**) N9 cells were directly or indirectly (N2a culture supernatant) exposed to SA as in Fig. 1A and viability was checked. Viability of N9 cells in both direct as well as indirect exposure group found to be 84–81% and 68–67% followed by 10 and 20 μM SA respectively. (**d**) N9 and N2a cells were also exposed to LPS (1 μg/ml) and no significant alteration in viability was recorded. (**e**) N2a viability following 24 h incubation with N9 culture supernatant with LPS was recorded to be around 83%. All the values in treatment groups were expressed as percent (%) to the respective control group. (**f**) Neonatal microglial purity was checked by CD11b immunostaining (**g**) purity was more than 96%. (**h**) Primary microglia were exposed to 5 μM SA and no significant mortality was observed. (**i**) Representative photomicrograph of microglia culture 24 h following 5 μM SA exposure. (**j**) Primary neurons were isolated and grown for 72 h in neurobasal medium and characterized with MAP2 as well as DCX antibody. (**k**) Viability of primary neurons following 5 μM SA exposure was recorded to be around 78%, whereas viability decreased to 58% following 5 μM SA exposed primary microglial culture supernatant. (**l**) Representative photomicrograph of primary neuron cultured either in exposure to SA or SA exposed microglial culture supernatant. Data shown in bar graphs represents Mean ± SE (Standard Error) of two-three independent experiments. ‘*p*’ denotes level of significance in comparison to control, *p < 0.05, **p < 0.01, ***p < 0.001, ns: non significant.

To determine how N9 culture supernatant affect the viability of N2a in the presence of SA, we have checked the effect of direct SA exposure as well as through culture supernatant on N9 and N2a cells. N2a cells were exposed to 10 and 20 μM of SA directly and incubated for 24 h. Similarly, another set of N2a cells were treated with 24 h microglial culture supernatant exposed to 10 and 20 μM of SA. N2a viability recorded in direct exposure to SA were 84% for 10 μM and 68% for 20 μM whereas N2a viability reduced drastically to 40% and 14% respectively when exposed to N9 culture supernatant for 24 h (Fig. 1a). Morphologically N2a reflected the unhealthy status following SA-exposed N9 culture supernatant (Fig. 1b). As the death in 20 μM of SA exposed N9 culture supernatant is very high, only 10 μM SA has been used in further experiments. Likewise, the viability of N9 cells was also evaluated 24 h following direct exposure to SA as well as N2a culture supernatant. Unlike N9 culture supernatant, N2a supernatant could not decrease the viability of N9 cells. In both the cases N9 viability found to be 84–81% and 68–67% followed 10 and 20 μM SA exposure respectively (Fig. 1c). Lipopolysaccharide (LPS) is a known activator of microglia. Therefore, LPS has also been used as a control in parallel to SA. N9 and N2a cells were also directly exposed to LPS (1 μg/ml) for 24 h and, in a separate experiment, N2a cells were also exposed to LPS-treated N9 culture supernatant followed by viability check by MTT assay. There was no alteration in the viability of the direct LPS exposure group for both N9 and N2a (Fig. 1d). Furthermore, 83% viability of N2a was retained upon exposure to the culture supernatant of LPS-treated N9 (Fig. 1e). Therefore, the culture of N9 cells in presence of SA turns the environment hostile for N2a cells.

To further validate our cell line data in primary cells we have performed bystander death of immature primary neurons in exposure to SA exposed primary microglia supernatant. Neonatal microglia isolated from 1-day old mouse pups were more than 95% pure as measured by CD11b immunostaining (Fig. 1f,g). Exposure to SA (5 μM) did not alter the viability of primary neonatal microglia significantly (Fig. 1h), which has also been supported by unaltered morphology (Fig. 1i). Primary neurons were isolated from embryonic day 16 mouse, cultured for 48 h and purity checked by immunostaining with MAP2 as well as DCX, a marker for developing neurons (Fig. 1j). The viability of primary neurons was recorded to be 98% in control group and 78% in 5 μM SA exposed group (Fig. 1k). The viability of immature primary neurons was not altered when exposed to control microglia culture supernatant, but the viability decreased to 58% when cultured with SA exposed microglia supernatant (Fig. 1k). SA exposed microglial culture supernatant inhibited neurite outgrowth and induced death (Fig. 1l). Therefore, a similar trend of bystander death of neurons has also been confirmed in immature primary neurons as shown in neuroblastoma cell line, N2a.

N2a and primary neurons isolated from embryonic day 16 mouse pups followed by 72 h culture in culture plate do not express glutamate receptor, NMDAR2B, which is a marker for mature neurons (Supplementary Fig. 1a). On the other hand primary neurons when cultured for a longer period (10 days) express NMDAR2B receptor indicating the neuronal maturation. They both express cystine/glutamate exchanger, xCT (Supplementary Fig. 1b).Therefore, we have used N2a cells as a model system of immature neurons.

### None of ROS, NO and proinflammatory cytokines are responsible for arsenic induces bystander death of immature neurons

**Figure 2:**
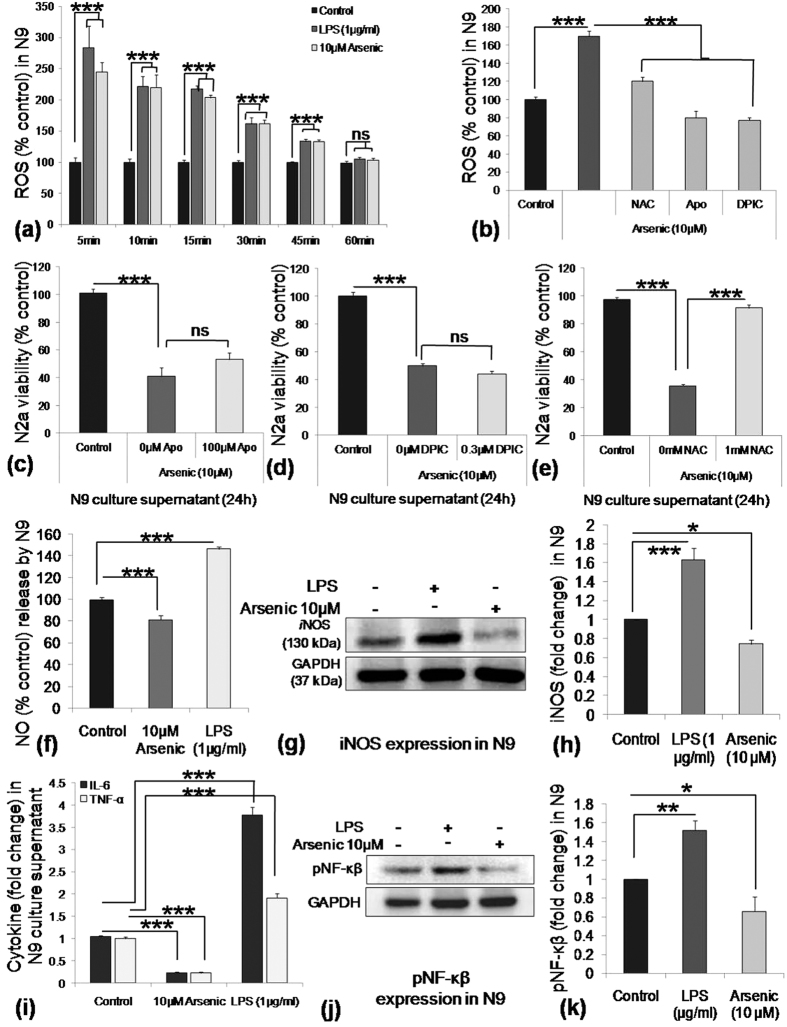
Effect of SA exposure on ROS, NO and proinflammatory cytokine (IL-6 and TNF-α) secretion by N9 cells and its effect on N2a viability. (**a**) N9 cells were treated with SA (10 μM) and LPS (1 μg/ml) in 96 well culture plate and immediately started measuring ROS initially every 5 min and later every 15 min until the ROS come down to control level. ROS level found to reach maximum around 2.5 fold in both LPS and SA treated group following 5 min of exposure. (**b**) N9 cells were pre treated with free radical scavenger, N-acetyl cysteine (NAC) and NADPH oxidase inhibitor, Apo and DPIC followed by SA treatment for 15 min. NAC, Apo and DPIC inhibited ROS generation. (**c**) N9 cells were treated 24 h with SA in presence of Apo (100 μM), DPIC (0.3 μM), NAC (1 mM) individually and supernatant collected. N2a cells were cultured 24 h in N9 culture supernatant and viability checked by MTT assay. (**c**) Apo and (**d**) DPIC could not rescue N2a, whereas. (**e**) NAC could restore the N2a viability. N9 cells were treated with 10 μM SA as well as 1 μg/ml LPS for 24 h followed by detection of NO and cytokine level in the culture supernatant. (**f**) LPS exposure significantly increased NO generation (150% of control) by N9 cells where as SA suppressed (70% of control) following 24 h incubation. (**g**) SA exposure also suppressed expression of iNOS, representative western blot and (**h**) densitometry graph of iNOS expression. (**i**) SA exposure suppressed secretion of proinflammatoy cytokines, IL-6 (0.19 fold to control) and TNF-α (0.25 fold to control), where as LPS increased the secretion of IL-6 and TNF- α. (**j**) N9 cells were harvested following 3 h treatment with either SA or LPS and phosphorylation status of NF-κβ was checked by western blot analysis, representative western blot and (**k**) densitometry graph of pNF-κβ. Data shown in bar graphs represents Mean ± SE (Standard Error) of two-three independent experiments. ‘*p*’ denotes level of significance in comparison to control, *p < 0.05, **p < 0.01, ***p < 0.001, ns: non significant.

To find out the reason behind bystander death of N2a induced by SA exposed N9 culture supernatant; we measured ROS levels in N9 cells. ROS level, measured by DCFDA reached more than 2.5 fold higher for the SA-exposed group compared to control at 5 min, which came down to basal level at 60 min. A similar trend was followed in LPS treated group (Fig. 2a). The addition of free radical scavenger, N-acetyl cysteine (NAC) brought down ROS to control level, whereas addition of NADPH-oxidase inhibitors, Apo and DPIC brought down the ROS level even below the control level (Fig. 2b). To check, whether the addition of various oxidative stress inhibitors could rescue N2a, N9 cells were cultured in presence of SA with or without various inhibitors and culture supernatant used to culture N2a cells followed by MTT viability test. Results showed that addition of apocynin (Apo) (Fig. 2c) and diphenyleneiodonium chloride (DPIC) (Fig. 2d) failed to rescue N2a cells from toxicity induced by N9 culture supernatant although both the inhibitors brought down the ROS level. Interestingly, NAC restored the viability to 87% (Fig. 2e). Therefore, the rescue of N2a by NAC points towards a different role of NAC other than its role as a free radical scavenger.

We explored factors other than ROS responsible for bystander death of N2a cells. We measured NO and proinflammatory cytokines (TNF-α and IL-6) as possible factors. SA exposure inhibited NO generation as well as iNOS expression. The level of NO was around 70% of control following SA exposure, whereas LPS (1 μg/ml) exposure enhanced NO generation to around 150% of control value (Fig. 2f). Western blot analysis showed that SA inhibited iNOS expression (Fig. 2g), and densitometric analysis revealed that iNOS expression was 0.7 fold in the SA-treated group, compared to control whereas LPS stimulated iNOS expression 1.6 fold (Fig. 2h). Likewise, SA also inhibited IL-6 and TNF-α secretion by N9 cells, on the contrary, exposure to LPS enhanced the secretion of both the proinflammatory cytokine, TNF-α and IL-6 (Fig. 2i). Phosphorylation of NF-κβ, is essential for iNOS, IL-6 and TNF-α decreased in the SA-exposed group in contrary to enhanced expression in the LPS-treated group (Fig. 2j). Densitometry analysis revealed that level of pNF-κβ in the SA-treated group was 0.6 fold compared to control, whereas around 1.5 fold increased the level upon LPS treatment (Fig. 2k). Therefore, SA affects NO and proinflammatory cytokine secretion by inhibiting its regulatory molecule iNOS and NF-κβ in N9 cells. Thus, pro-inflammatory factors are not responsible for bystander death of developing neurons.

### SA induced cellular GSH synthesis in microglia and helps its survival

**Figure 3:**
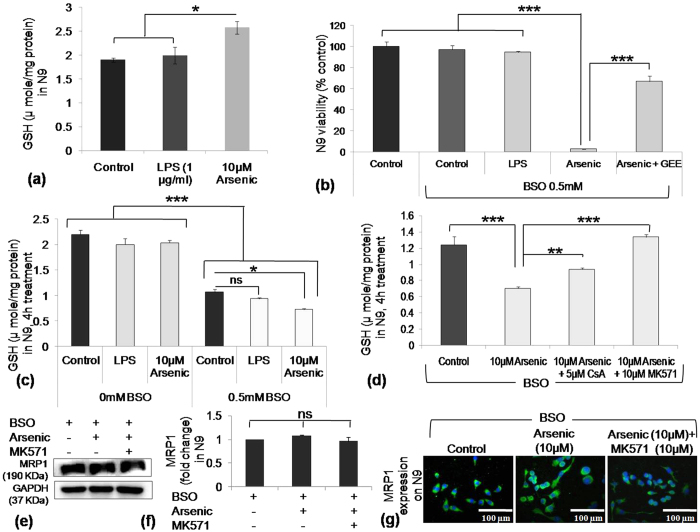
Effect of SA exposure on GSH level in N9 cells and involvement of MRP1 in depleting cellular GSH. (**a**) N9 cells were exposed to SA as well as LPS for 24 h and cellular GSH level measured. SA exposure increased cellular GSH level to 2.5 μmol/mg protein significantly higher than control where as insignificant alteration observed following LPS exposure. (**b**) N9 cells were exposed to SA as well as LPS in presence of BSO for 24 h, one SA treated group was supplemented with exogenous GSH ethyl ester (15 mM). Viability checked following 24 h incubation. BSO did not affect viability of control as well as LPS treated N9 cells but severely compromised the viability of SA exposed group (≈7%). Supplementation of exogenous GSH increased N9 viability to 66%. (**c**) N9 cells were exposed to SA as well as LPS and cultured 4 h in presence or absence of BSO. Following 4 h incubation with BSO, level of cellular GSH decreased almost 50% in all the groups compared to BSO unexposed group. (**d**) N9 cells were exposed 4 h to SA in presence of BSO with or without MRP1 inhibitor, Cyclosporin-A (CsA) and MK571. Following 4 h of incubation, cellular GSH depleted in SA treated cells (0.7 μmol/mg protein) significantly more than the control (1.2 μmol/mg protein) cells whereas addition of CsA and MK571 inhibited GSH (0.94 and 1.3 μmol/mg protein respectively) depletion. N9 cells were seeded in 6 well culture plate and treated 4 h as in figure d western blot analysis for the expression of MRP1, (**e**) representative western blot and (**f**) densitometric graph of MRP1 expression. (**g**) Similarly, N9 cells were seeded on cover slips and treated 4 h as in figure D followed by immunostaining to check the MRP1 expression level. Data shown in bar graphs represents Mean ± SE (Standard Error) of two-three independent experiments. ‘*p*’ denotes level of significance in comparison to control, *p < 0.05, **p < 0.01, ***p < 0.001, ns: non significant.

We searched for the factors responsible for bystander neuronal death and found none of ROS, NO, IL-6 or TNF-α to be responsible. Therefore, we checked GSH level in N9 cells. SA-treatment for 24 h caused a significance increase (1.3 fold to control); while LPS had no effect on GSH level of N9 cells (Fig. 3a). Inhibition of GSH synthesis by BSO (0.5 mM) for 24h did not affect the viability of control as well as LPS treated N9 cells, whereas, BSO severely compromised viability (~3%) of N9 cells in SA treated group. However, exogenous supplementation of glutathione derivative, glutathione ethyl ester (GEE, 15 mM) rescued the viability (~66%) of the BSO-treated, SA exposed N9 cells (Fig. 3b). In presence of BSO, cells failed to synthesize GSH and therefore, N9 cells undergo severe death which is rescued by exogenous GEE supplementation. To check the requirement of GSH synthesis in LPS and SA treated group, N9 cells were treated 4 h with LPS and SA in presence or absence of BSO followed by estimation of cellular GSH level. We have selected 4 h time point because, in presence of BSO and SA, N9 cells start dying beyond this time point. Cellular GSH level in the BSO-treated group went down to almost half the concentration of BSO untreated cells (2 μmol/mg protein) (Fig. 3c), Whereas GSH level in SA with BSO-treated group decreased even more than BSO-treated control and LPS group (Fig. 3c). To find out the reason behind the reduced GSH in SA treated group in the presence of BSO, these cells were incubated with 5 μM MRP1 inhibitor, Cyclosporin-A (CsA, 5 μM) and MK571 (10 μM). Interestingly, in the presence of CsA and MK571, GSH level increased significantly compared to only arsenic exposed group (Fig. 3d). We have also checked the expression of MRP1 and found no significant changes 4 h following SA exposure; thereby confirming that the alteration of cellular GSH in different groups of treatment is not due to the change in MRP1 expression level (Fig. 3e–g). Therefore, arsenic exposure induced N9 cells to synthesize GSH which helps it to survive stress induced by SA.

### Exogenous supplementation of cystine in microglial culture supernatant rescued immature neurons from bystander death

**Figure 4:**
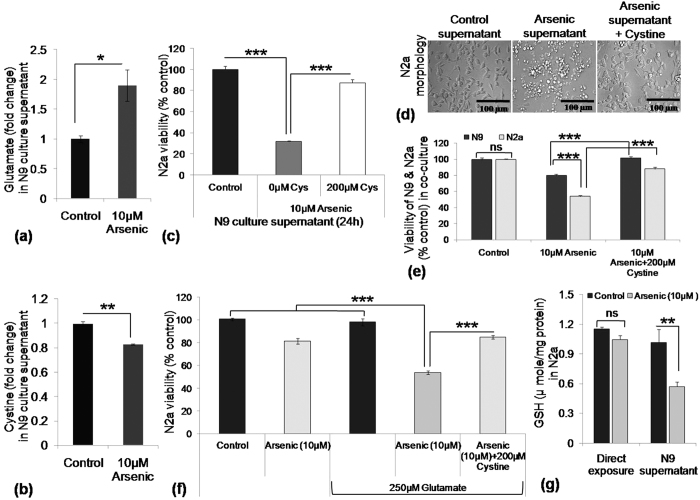
Effect of SA exposure on cystine/glutamate level in N9 culture supernatant and effect of exogenous cystine supplementation on recovery of N2a from bystander death. N9 cells were exposed to SA for 24 h in 96 well culture plate, culture supernatant collected and measured for (**a**) Glutamate and (**b**) Cystine content. In exposure to SA, glutamate content in the culture supernatant was increased (1.89 fold), in contrary cystine level was decreased (0.6 fold) significantly. N2a cells were cultured in cystine (200 μM) supplemented SA exposed N9 culture supernatant for 24 h and viability was checked. (**c**) Cystine supplementation rescued N2a significantly from bystander death, viability increased from 31% in SA-exposed group to 90.6% in cystine supplemented group. (**d**) Representative photomicrographs of experiment (**c)**. (**e**) Effect of SA exposure and recovery by cystine supplementation was also evaluated in N9/N2a co-culture. Viability of N2a cells was recorded to be significantly lower (54%) than N9 (80%) cell in co-culture. Following exogenous supplementation of cystine viability of both cells increased significantly. (**f**) The effect of high level of glutamate on N2a cells and the reversibility of the effect by cystine supplementation has also been tested. N9 cells were treated with SA, glutamate and cystine separately as well as co-treated as in figure (**c**). Addition of 250 μM of glutamate (actual value in the culture supernatant was around 265 μM) to SA treated group significantly decreased N9 viability (53%) compared to only SA treated group (88%). Supplementation of 200 μM cystine to SA-glutamate co-treated group recovered N2a viability to 78.7%. (**g**) Status of GSH level in N2a cell was checked 12 h following direct SA exposure and N9 culture supernatant. GSH level was decreased significantly in N9 supernatant exposed N2a cells (0.57 μmol/mg protein) compared to direct SA exposed N2a cells (1.0 μmol/mg protein), whereas no significant alteration observed in unexposed group. Data shown in bar graphs represents Mean ± SE (Standard Error) of two-four independent experiments. ‘*p*’ denotes level of significance in comparison to control, *p < 0.05, **p < 0.01, ***p < 0.001, ns: non significant.

We have shown that N9 cells synthesize GSH. Therefore, we assessed the concentration of cystine and glutamate in the culture supernatant. Following 24 h incubation, in the presence of SA, glutamate level increased about 1.89 fold (Fig. 4a) and cystine level found to be 0.6 fold (Fig. 4b) of control in the N9 culture supernatant. We hypothesized that depletion of cystine and increase of glutamate level in the N9 culture supernatant could be the probable reason for N2a death; therefore, N9 cells were cultured 24 h in exposure to SA supplemented with 200 μM cystine. When grown in this culture supernatant, the viability of N2a increased from 31% to 91% (Fig. 4c). Morphologically also, N2a cells reflected the recovery of normal status like control following cystine supplementation (Fig. 4d). The viability of N9 and N2a, as well as their recovery by cystine supplementation, were also evaluated in co-culture, which mimics the *in-vivo* condition to some extent. The viability of N9 and N2a in co-culture was found to be around 80% and 54% respectively; the pattern was similar to that of individual culture (Fig. 4e). Following cystine (200 μM) supplementation viability increased around 101% and 88% respectively for N9 and N2a (Fig. 4e). As there was decreased cystine and increased glutamate concentration in the extracellular environment, we checked whether competitive inhibition of cystine transport playing any role in N2a death in the arsenic environment. Glutamate level in the N9 culture supernatant following 24 h arsenic exposure was measured to be around 265 μM. Therefore, we wanted to check whether increased glutamate level causes any toxicity in the presence of arsenic in N2a cells. We used 250 μM, little less than the exact measured value, glutamate for the experiment. Glutamate (250 μM) or SA (10 μM) alone did not affect N2a viability significantly but, when co-exposed decreased N2a viability to 50%, which was rescued significantly (80%) by the addition of extra cystine (200 μM) (Fig. 4f). Effect of direct and indirect SA exposure on GSH level of N2a was checked. There was no significant alteration in cellular GSH level followed by direct SA exposure to N2a; on the other hand, treatment with SA-exposed N9 culture supernatant reduced the GSH level in N2a (0.57 μmol/mg protein) compared to respective control (1.02 μmol/mg protein) (Fig. 4g). Therefore, arsenic-induced synthesis of GSH by N9 cells altered the cystine/glutamate concentration in the culture medium and thereby induces bystander death of N2a.

### SA exposure increased expression of xCT, as well as Nrf2 in microglia

**Figure 5:**
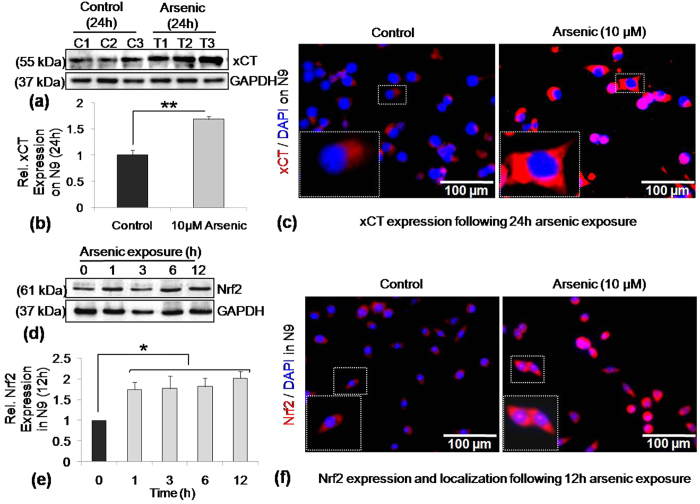
Effect of SA exposure on xCT and its regulatory molecule, Nrf2 in N9 cells. Western blot analysis detected significantly increased expression (1.7 fold) of xCT following 24 h SA (10 μM) exposure. (**a**) Representative western blot of xCT, (**b**) Densitometric analysis of xCT expression, (**c**) Representative photomicrograph (40X) of immunostained xCT. N9 cells were harvested following exposure of SA for different periods (0, 1, 3, 6 and 12 h), lysed and subjected to SDS-PAGE and probed with Nrf2 antibody. SA exposure increased the Nrf2 level at 1 h and maintained 1.7–1.9 fold higher expression level until 12 h of incubation. (**d**) Representative western blot of Nrf2, (**e**) Densitometric analysis of Nrf2 expression, (**f**) Representative photomicrograph (40X) of immunostained Nrf2 (Red) following 12 h SA exposure. Data shown in bar graphs represents Mean ± SE (Standard Error) of three-four independent experiments. ‘*p*’ denotes level of significance in comparison to control, *p < 0.05 or **p < 0.01.

Cystine and glutamate transport for synthesizing GSH, is mediated through xCT. Therefore, expression levels of both xCT and its redox-sensitive transcription factor, Nrf2 were checked by Western blot analysis as well as immunocytochemistry. Expression of xCT, detected by western blot analysis, increased around 1.7 fold compared to control following 24 h of SA exposure in N9 cells (Fig. 5a,b). Immunocytochemistry data verified the increase in xCT expression (Fig. 5c). Western blot analysis of Nrf2 expression detected 1.7–1.9 fold increase following 1 h SA exposure and maintained the higher level until 12 h (Fig. 5d,e). Likewise, immunocytochemical staining of Nrf2 also showed increased expression as well as enhanced nuclear localization 12 h following arsenic exposure (Fig. 5f). Therefore, arsenic exposure increases Nrf2 expression as well as nuclear localization along with a rise in xCT expression in N9 cells.

### Inhibition of microglial xCT by siRNA rescue N2a from bystander death induced by arsenic exposed microglial culture supernatant

**Figure 6:**
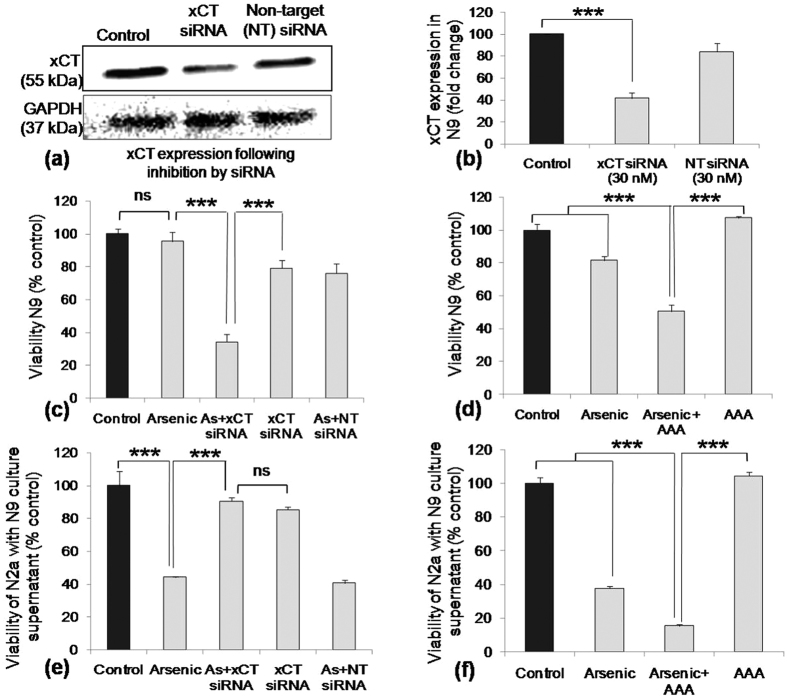
Role of xCT in SA induced alteration in N9 viability and N2a bystander death. To confirm the role of xCT in SA induced N9 and N2a viability, N9 cells were transfected with siRNA and following 36 h xCT expression was checked. The xCT expression (42%) was observed to be decreased significantly compared to control, (**a**) representative western blot of xCT and (**b**) densitometric analysis of xCT western blots. (**c**) To evaluate the role of xCT in N9 viability, cells were transfected with siRNA followed by SA exposure. Viability of siRNA and SA co-treated cell was recorded to be 34%, significantly lowered than control. (**d**) Similar pattern was recorded in AAA treated group, where SA and AAA co-treated group showed lowest viability around 51%. (**e**) Bystander death of N2a induced by siRNA and SA co-treated N9 culture supernatant was evaluated. Only SA treated supernatant and NT siRNA-SA co-treated N9 supernatant induced significant N2a death (44% and 41% viability respectively) compared to control, but viability of N2a exposed to xCT siRNA-SA co-treated as well as only xCT siRNA treated supernatant was not altered significantly compared to control. (**f**) Similar pattern of N2a bystander death induced by AAA treated N9 culture supernatant was observed except SA-AAA co-treated group. Viability of N2a in exposure to SA-AAA co-treated N9 supernatant (15%) showed reverse pattern compared to SA-siRNA treated group (90%). Data shown in bar graphs represents Mean ± SE (Standard Error) of two-three independent experiments. ‘*p*’ denotes level of significance in comparison to control, *p < 0.05, **p < 0.01, ***p < 0.001, ns: non significant.

To confirm the role of xCT in the viability of microglia and bystander death of immature neuron in the presence of arsenic, xCT in microglia were inhibited by siRNA as well as small molecule inhibitor aminoadipic acid (AAA). Transfection with siRNA inhibited around 60% of xCT expression in N9. On the other hand, xCT expression was detected to be around 84% in non-target (NT) siRNA transfection group, (Fig. 6a,b). When siRNA inhibited N9 cells were exposed to SA, the viability of siRNA and SA co-treated cells was decreased by 34% in comparison to control. The viability of only SA, siRNA and SA-non-target siRNA co-treated cells were recorded to be 95%, 79% and 76% respectively (Fig. 6c). Similar pattern or N9 viability was observed following xCT inhibitor, AAA (Fig. 6d) with the viability of SA and AAA co-treated cells recorded to be lowest, around 51%. Arsenic and siRNA treated microglial culture supernatant induced bystander N2a death. The viability of N2a in exposure to only arsenic and non-target siRNA treated microglial supernatant was found to be around 44% and 41% respectively (Fig. 6e). Interestingly, SA-siRNA co-treated microglial supernatant did not affect the viability of N2a (90%). Only xCT siRNA treatment neither affected the viability of N9 or bystander death of N2a (Fig. 6e). A similar pattern of N2a bystander death was observed following AAA treated microglial supernatant exposure, except SA-AAA co-treatment group. The viability of N2a following exposure of SA-AAA co-treated microglial supernatant was recorded to be 15%, whereas SA-siRNA co-treated group showed around 90% viability (Fig. 6f). The difference in N2a viability following exposure to microglial supernatant may be due to the presence of AAA in the culture supernatant and subsequent inhibition of xCT in N2a cells.

### Gestational exposure of SA increased xCT expression in microglia, altered extracellular cystine/glutamate level, induced death of immature neurons and the effect was restored by NAC supplementation

**Figure 7:**
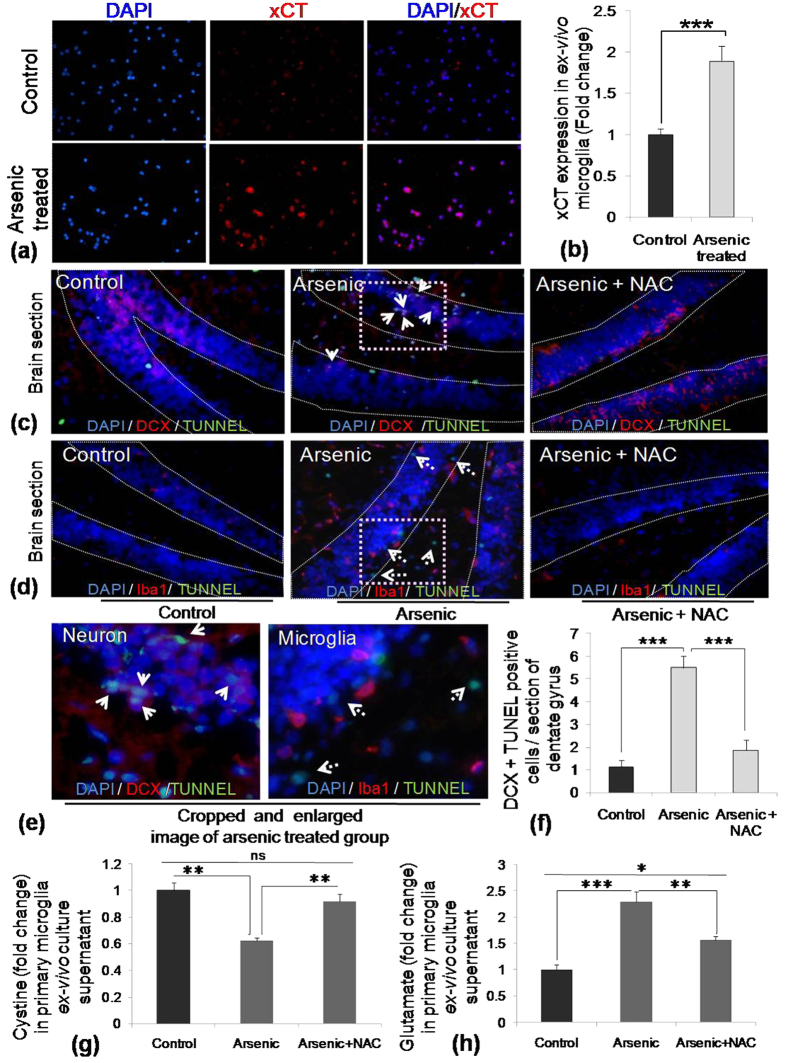
Effect of *in-vivo* SA exposure and NAC supplementation on viability of neurons and microglia in dentate gyrus of developing brain. Pregnant Balb/c mice (2 pregnant mice for each group) were exposed to 0.38 mg/kg body wt of SA, one set of SA exposed female were co treated intraperitoneally with NAC (10 mg/kg body wt) every alternate day starting from Gestational day (GD) 0 until weaning of the pups (PND 22–25). Animals were sacrificed and brain tissue processed for *ex-vivo* microglia culture and cryo-sectioning. Effect of *in-vivo* SA exposure xCT expression in microglia and microglia induced alteration in cystine/glutamate level. Primary microglia seeded on glass cover slips and immunostained with xCT antibody. (**a**) representative photomicrograph, (**b**) quantitative data of xCT expression. (**c**) Co-immunofluorescence staining DCX and TUNEL revealed more apoptotic DCX positive cells in SA exposed pups compared to control and arsenic + NAC treated pups brain sections (DCX positive apoptotic cells: arrow heads). (**d**) Immunofluorescence staining with Iba1 showed increased microglia number following SA exposure than control and arsenic + NAC treated brain sections. TUNEL immunofluorescence was visible in SA exposed Iba1 stained slides but none were merged with Iba1. (**e**) Sections of arsenic treated groups were cropped and enlarged to show the overlapping of TUNEL with DCX and Iba1. Photomicrographs were taken at 40X. (**f**) Quantitative analysis of TUNEL assay in brain cryosections were represented as DCX/TUNEL positive cells per section showing significant increase in arsenic exposed group as compared to control and arsenic + NAC group. To check alteration in cystine/glutamate level, Primary microglia were cultured from control and treated pups followed for 18 h. Culture supernatant collected and measured for cystine and glutamate (**g**) Cystine level decreased in SA-exposed group whereas NAC treatment restored the level near to control value. (**h**) On the other hand, glutamate level increased following SA exposure and NAC treatment brought down the Glutamate level significantly. N = 4 pups (2 from each of two pregnant female of each treatment group). ‘*p*’ denotes level of significance in comparison to control, *p < 0.05, **p < 0.01, ***p < 0.001, ns: non significant.

SA exposure *in-vitro* increased xCT expression in N9 cells. To further validate SA-induced alteration in xCT *in-vivo*, primary microglial cells were isolated from control and gestationally exposed 22 days old pups and xCT expression checked. Gestational arsenic exposure was found to induce around 2 fold xCT expression in primary microglia (Fig. 7a,b). Our *in-vitro* finding of microglia-mediated bystander death of immature neurons in exposure to SA was further validated *in-vivo*. Brain sections from control and treated pups were co-immunostained with TUNEL and DCX, for immature neurons as well as TUNEL and Iba1, for microglia. SA exposure induced death of DCX positive cells in the dentate gyrus region of developing brain. Co-administration of NAC (10 mg/kg body wt) along with SA treatment inhibited the cell death (Fig. 7c,e). TUNEL positive cells were also visible in Iba1 stained slides, but TUNEL fluorescence did not merge with Iba1 fluorescence (Fig. 7d). Interestingly SA exposure increased Iba1 positive cells in dentate gyrus. NAC supplementation helped decrease the TUNEL positive cells as well as brought back the number of Iba positive cells to control level (Fig. 7d,e). Quantitative analysis of DCX and TUNEL positive cells (Fig. 7c,e) showed recovery in neuronal viability following NAC supplementation (Fig. 7f). Therefore, developing/immature neurons are more susceptible to gestational exposure of SA. Similarly, *in-vitro* alteration in cystine/glutamate level was also validated in the *ex-vivo* culture supernatant of primary microglia isolated from gestationally exposed mice. Primary microglia were isolated, cultured *ex-vivo* for 18 h and collected for measuring cystine and glutamate level. Cystine level was decreased (0.6 fold to control) in the supernatant of cells isolated from SA-treated pups where as cystine level increased near to control level (0.9 fold to control) in the supernatant of cells isolated from SA and NAC co-treated pups (Fig. 7g). In contrary glutamate level in *ex-vivo* culture, supernatant showed opposite pattern to cystine, higher glutamate (around 2.39 fold to control) in SA treatment group, which has been decreased from 2.3 fold to 1.5 fold following NAC co-treatment with SA (Fig. 7h). Thus, an *in-vitro* SA-induced increase in xCT, as well as alteration in cystine/glutamate level, had been validated in *ex-vivo* following gestational SA exposure.

## Discussion

Arsenic exposure has been reported to reduce learning and memory performance^10^,^26^. It has also been predicted to cause neurodegenerative diseases^8^,^27^. On the other hand, neurodegeneration has been closely associated with the altered activity of microglia, which is the fastest glial cell type to respond to any toxic insult^28^. Therefore, the present study is focused on deciphering the mechanism of microglia-induced bystander death of immature neurons in the presence of arsenic. Arsenic-mediated downregulation of glutamate receptors on mature neurons supports adaptability towards excitotoxic effects^29^, whereas, newly generated immature neurons in developing brain lacks glutamate receptors, therefore, does not show excitotoxicity and also found to be most sensitive to oxidative stress^20^. This fact has driven us to study immature neurons which have been a primary target of arsenic; however the mechanisms are poorly understood. Studies with benzopyrene and lead showed microglia induces bystander neuronal death by releasing cytotoxic factors like ROS, NO, TNF-α and IL-6^30^,^31^. We showed that microglia-mediated bystander death of immature neurons in an arsenic environment was induced by none of ROS, NO, TNF-α and IL-6. We evince that microglia in the presence of arsenic alters extracellular cystine and glutamate level, which plays a significant role in this event.

We showed that arsenic-treated microglial culture supernatant induced death of N2a cells. Conversely neuronal culture supernatant had no effect on the microglial viability pertaining to its less vulnerability to the environmental condition^19^. There are sporadic reports of increased level of neurotoxic factors like NO^32^ and proinflammatory cytokines (TNF-α, IL-6)^33^ in human blood samples as well as experimental mice liver following arsenic exposure^34^,^35^. In the present study 10 μM *in-vitro* arsenic exposure severely suppressed TNF-α as well as IL-6 secretion along with a reduction of NO in the microglial culture supernatant, which is in agreement with other published reports^36^. Suppression of iNOS, NO, TNF-α or IL-6 may have resulted from the inhibition of phosphorylation of NF-κβ by arsenic^37^. Thereby, ruling out the possibility of NO and proinflammatory cytokine-induced bystander death of immature neurons.

Inorganic arsenic activates NADPH oxidase^38^,^39^ and thus induces ROS generation, which may, in turn, evoke cell death^30^. In the present study inhibition of NADPH oxidase activity in microglia could not rescue N2a cells. On the other hand, LPS treated microglial culture supernatant does not compromise N2a viability; even though stimulate ROS in N9 cells. These two facts prove non-involvement of ROS in N2a death^40^. Rescue of N2a cells by NAC supplementation in microglial culture indicates the involvement of GSH in the survival of N2a from microglia-induced bystander death^41^.

Arsenic-induced alteration in GSH metabolism adversely affects the brain environment^42^,^43^. Microglia maintains its viability in the presence of arsenic by increased synthesis of GSH^25^,^44^ which helps to efflux arsenic as As-[GS]3 through MRP1^24^,^25^,^45^. Therefore, the involvement of MRP1 has been confirmed by inhibition of GSH efflux following addition of Cyclosporin-A as well as MK571^46^. Interestingly LPS-induced stress on microglia is counteracted by the conversion of GSSG-GSH using NADH. While synthesizing GSH, microglia intake cystine in exchange for glutamate through xCT in developing brain^16^. Our study may be helpful explaining the result of an earlier study showing an increase in glutamate levels in the brain hippocampus upon SA exposure^47^, Which may be produced from arsenic-induced nrf2 mediated increased expression of microglial xCT. Microglial culture supernatant containing arsenic, lower cystine and increased glutamate level induces bystander death of N2a, which is rescued by cystine supplementation^20^. Higher Glutamate levels in microglial culture supernatant contribute towards N2a cell death by competitively inhibiting xCT present on immature neurons in the developing brain, which is low in basal cellular GSH level^19^.

The role of microglial xCT in the survival of immature neurons during arsenic stress has also been evaluated by xCT inhibition study using siRNA as well as system Xc inhibitor, AAA. Partial inhibition of microglial xCT by siRNA as well as AAA induced death of microglia in the presence of arsenic. Small interfering RNA (siRNA)-inhibited, arsenic-exposed microglial culture supernatant did not cause significant bystander death of N2a, Whereas AAA-inhibited, arsenic-exposed microglial culture supernatant induced bystander death of N2a. This opposite bystander death pattern of N2a occurred because of the presence of AAA in microglial culture supernatant which in turn inhibits neuronal xCT, thereby inducing neuronal death. Interestingly, inhibition of xCT by siRNA or AAA neither affected N9 nor induced bystander death of N2a in the absence of arsenic. Thus, microglial xCT plays a significant role in survivability of immature neurons in a stressed condition.

**Figure 8:**
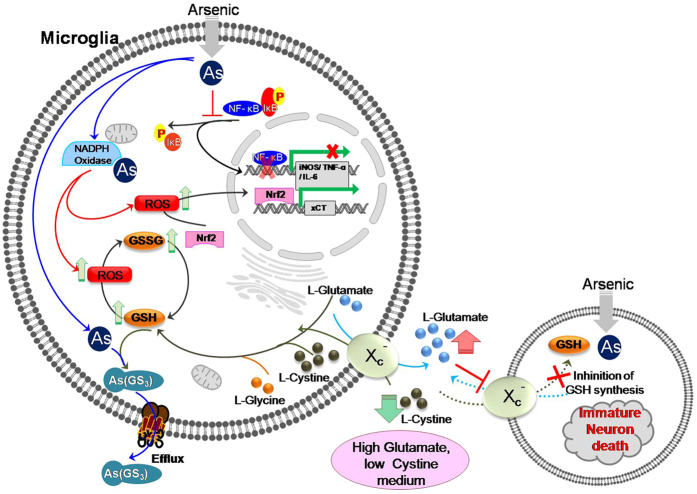
Role of microglia in mediating bystander death of developing neurons in arsenic environment. SA once entered into microglia (i) inhibits phosphorylation of NF-κβ thereby inhibiting expression of iNOS, TNF-α and IL-6 (ii) induces ROS which in turn enhance expression of xCT through induction of Nrf2, (iii) induces cellular GSH synthesis, which is used to efflux arsenic as GSH conjugate (As[GS]_3_). Induction of cellular GSH synthesis by arsenic requires intake of cystine in exchange of glutamate expelled out through xCT. Thus, microglia lower cystine from extracellular environment and at the same time accumulates glutamate. In this environment viability of developing neurons affected in two ways, in one hand low cystine level limits the supply for GSH synthesis, on the other hand higher glutamate level block the route of supply by inhibiting xCT, thereby inducing bystander death of developing neuron.

There have been contradictory *in-vivo* study reports regarding the role of xCT in neuronal survivability. Bundel *et al*.^48^, showed xCT knockout mouse had no brain atrophy, whereas brain atrophy was observed in subtle gray (sut/sut) mice containing nonfunctional xCT^15^,^48^. Till date, there has been no study describing the impact of alteration in microglial glutathione on the immature neuron. Immature neurons are the most vulnerable to toxic insult like early life arsenic exposure. Owing to its high density and high xCT activity in hippocampus microglia plays a significant role in altering the viability of immature neurons^49^,^50^. Our *in-vitro* finding of bystander death of immature neuron was validated *in-vivo*. Gestational exposure to arsenic-induced death of immature neurons as revealed by co-labeling of brain sections of 22-day old pups with doublecortin (DCX), a marker for immature neurons in developing brain and TUNEL, which is in agreement with a previous study^51^,^52^. Alteration in the viability of developing neurons, in turn, may affect learning as well as spatial working memory response^53^. NAC supplementation rescue DCX positive cells in our study by promoting GSH metabolism in the brain^41^. Increased expression of xCT in primary microglia isolated from arsenic-exposed mice as well as decreased level of cystine in *ex-vivo* culture supernatant and its restoration in NAC support increased number of microglia in the dentate gyrus of developing neurons with higher cystine demand. Increased cystine demand, subsequently causing increased glutamate level in the local milieu, which has been evident in our *ex-vivo* glutamate measurement, and thereby compromise neuronal viability. Glutamate-mediated oxidative toxicity can also be enhanced by the arsenic-induced reduction in astroglial population in developing brain as well as a decrease in levels of glutamate uptake transporters (EAAT1 and EAAT2)^54^,^55^,^56^. Taken together, we show, in the presence of arsenic microglia lowers cystine and consequently increases glutamate level in the extracellular milieu which in turn competitively inhibits xCT mediated cystine uptake, resulting in reduced GSH leading to bystander death of developing neurons (Fig. 8). Thus, the study helps to understand the role of microglia in modifying extracellular environment which in turn affect neuronal health. Although the role of xCT in neurodegeneration and neuroprotection remains paradoxical, arsenic-induced upregulation suggests its indispensability for survival in stress condition. Therefore, the present study reveals that while surviving arsenic stressed microglia alters extracellular cystine/glutamate balance which leads to the death of immature neurons.

## Methods

### Reagents and Antibodies

Sodium arsenite (SA) (NaAsO2), Percoll, cell culture medium (DMEM/F12), antibiotics, papain, Bacterial lipopolysaccharide (LPS), sodium dodecyl sulphate (SDS), acrylamide, bis-acrylamide, Tris–HCl, MTT, MK571, N-acetyl cysteine (NAC), dimethyl sulphoxide, bromophenol blue, Tween 20, β-mercaptoethanol, N-ethylmaleimide, o-phthalaldehyde, EDTA, protease inhibitor cocktail, glycerol, 10× Western blocking buffer, cystine, O-phthalaldehyde (OPA), 9-fluorenylmethyl chloroformate (FMOC-Cl), 3-mercapto propionic acid (MPA), L-Cystine amino acid standard, BSTFA + TMCS, 99:1, sodium phosphate monobasic monohydrate, sodium hydroxide, boric acid, acetonitrile (LC grade), methanol (LC grade), and n-hexane were obtained from Sigma (St. Louis, MO, USA). Fetal bovine serum (FBS) was purchased from Cell Clone Inc. Antifade mounting medium, N-2 supplement, and Alexa fluor antibodies were obtained from Life Technologies. 2′, 7′-dichlorofluorescein diacetate (DCFH-DA) were purchased from Molecular Probes Inc. (Eugene, Oregone). PVDF membrane and chemiluminescence substrates were obtained from Merck-Millipore (USA). Apocynin (Apo) and cyclosporine-A (CsA) were purchased from TCI chemicals. Diphenyl Iodonium Chloride (DPIC) received from Caymann, San Diego, CA, USA. TUNEL kit was procured from Roche. Doublecortin (DCX) and Iba1 antibody were purchased from Abcam, Cambridge, England and Merck-Millipore (USA). pNF-Кβ antibody was obtained from cell signaling technology, Beverly, MA, USA. xCT antibody was purchased from Pierce Biotechnology, Waltham, Massachusetts, USA. Nrf2 antibody and HRP conjugated secondary antibodies were obtained from Santa Cruz, CA, USA. Chromatographic-grade water was produced by a Milli-Q system (Millipore, Billerica, MA). siRNA for xCT and siPORT NeoFX transfect agent were procured from Ambion, Texas, USA.

### Animals and treatment

Six to eight week old male and female Balb/c mice were procured from the CSIR-Indian Institute of Toxicology Research (CSIR-IITR) animal facility. All The protocol for the study was approved by the Institutional Animal Ethics Committee of CSIR-IITR, Lucknow, India, and all experiments have been carried out in accordance with the guidelines laid down by the committee for the purpose of control and supervision of experiments on animals (CPCSEA), Ministry of Environment and Forests (Government of India), New Delhi, India. Mice were housed at 25 °C with food and water supplied ad libitum. Following mating, females were divided into three groups; control group, arsenic treatment group, and SA-NAC co-treatment group. Three females were taken for each group and kept in a separate cage. Pregnant females were gavage fed daily with 0.38 mg/kg body weight from gestational day 5 (GD5) until the weaning period of pups (Post Natal Day 22–25). The control group received water. Treatment with N-acetyl cysteine (NAC) was given intraperitoneally (10 mg/kg body weight) every alternate day^57^.

### Cell culture

Mouse microglia cell line, N9 and neuroblastoma cell line, Neuro-2a (N2a) used in the present study, were the kind gift from Dr. Anirban Basu, National Brain Research Center (NBRC), India. N9 cell line is widely used as an *in-vitro* model, representing similar features of primary microglia^58^. N2a does not express glutamate receptor^59^, therefore widely used as an *in-vitro* model of neuronal differentiation^60^. Cells were cultured in DMEM/F12 in the presence of 10% FBS at 37 °C in an incubator maintaining 5% CO2. N2a cells were used for experiments up to 5–6 passage following thawing. For experimental purpose cells were grown in 96 well or 6 well, culture plates overnight followed by treatment with SA or LPS.

### Isolation of primary microglia (neonatal and postnatal) and culture

Neonatal microglia was isolated from pups (PND1) according to the protocol developed by Saura *et al*.^61^. Briefly, cerebral cortices were separated, and meninges were removed in ice-cold HBSS. The cell suspension was plated in 24 well plates, and confluent cultures were treated with DMEM/F12: Trypsin (0.25%) in 1:1 ratio for 30–45 min. Following incubation primary astrocytes will detach from the plate surface and microglial cells will remain attached on the surface of culture plate. Isolated primary neonatal microglia were seeded in a 24 well culture plates (60,000 cells/600 μl complete culture medium/well) and incubated for 24 h in presence and absence of arsenic. Microglia culture supernatant was collected and stored for bystander death assay and measurement of cystine and glutamate. Primary microglia were also isolated from the brain of postnatal mouse aged 22–25 days by percoll gradient and cultured in DMEM/F12 supplemented with 2% FBS and N-2 supplement for 18 h^4^.

### Isolation and culture of immature primary neurons

Primary immature neuron culture prepared according to the protocol developed by Shih *et al*. 2003. Pregnant BALB/c mice were sacrificed and dissected carefully to remove embryos (embryonic day 16). Cerebral cortices were mechanically dissociated in cold HBSS and incubated in 0.05% trypsin/EDTA at 37 °C for 10 min, followed by centrifugation at 1000 rpm for 10 min. Cells were resuspended in neurobasal medium containing B-27 supplement (Invitrogen, Carlsbad, CA), L-glutamine (0.5 mM), penicillin (100 units/ml) and Streptomycin (100 μg/ml). Cells were seeded on poly-L-lysine-coated chamber slides and 96 well plate and grown in a CO2 incubator at 37 °C with 5% CO2 for 48 h.

### Determination of cell viability

Cell viability was determined using MTT (4,5-dimethylthiazol-2-yl-2,5 diphenyltetrazolium bromide) following standard protocol^62^. Briefly, N9 and N2a cells were seeded in 96 well plates at a density of 10,000 cells/100 μl/well, treated with SA, LPS or culture supernatant for 24 h. Two hours before completion of incubation 10 μl of MTT (5 mg/ml) added to each well and allowed for formazan crystal formation. Crystals were dissolved in dimethyl sulfoxide (DMSO), and absorbance was read in a microplate reader (Ώ fluostar, BMG labtech) at 570 nm. Absorbance values were converted to the percentage of control values and represented as percent viability.

### Bystander neuronal death assay

To assess bystander death of immature neurons, induced by microglia following SA treatment, microglia were seeded in 96 well cultures plate (N9 at a density of 10,000 cells/well/100 μl and primary microglia at a density of 60,000 cells/300 μl). Cells were grown overnight and treated with SA (N9 were exposed to 10 and 20 μM of SA for 24 h, primary microglia were exposed to 5 μM SA). N9 culture supernatant added to N2a, and primary microglia supernatant added to primary immature neuron culture followed by further 24 h incubation. MTT dye was added 2 h before completion of treatment time and processed for viability test^30^.

### Co-culture survival assay

20,000 N9 and N2a cells were co-cultured in 24 well plates with insert (0.4 μm) (N9 on 24 well surface and N2a in a transwell insert) and incubated overnight. Cells were treated with arsenic in a serum free medium for 24 h. The culture supernatant collected and added in fresh N9-N2a co-culture as described previously for another 24 h. This procedure was followed till 96 h. The viability of N9 and N2a co-cultured for 96 h was assessed by MTT assay.

### siRNA Transfection

N9 cells were seeded in a six-well (0.5 × 106 cells/well) and 96 well plates (10,000 cells/well) respectively for western blot analysis and arsenic treatment. Cells were treated with 30 nM xCT siRNA (Ambion) for 24 h followed by replacement with serum free DMEM/F12 media for additional 12 h. Expression of xCT was confirmed by western blot analysis. Following transfection cells were treated with arsenic (10 μM) for 24 h and culture supernatant were collected for bystander death.

### Glutamate and cystine estimation in culture supernatant using GC-MS and UHPLC

N9 culture supernatant were prepared by treating 10,000 cells with arsenic for 24 h in 96 well culture plates. Control as well as arsenic-treated supernatant collected and measured for cystine content by UHPLC and glutamate content by GC-MS. *Ex-vivo* culture supernatant prepared by isolating primary microglia from control as well as treated pups and incubating 1 × 10^5^ cells/100 μl/well in 96 well culture plates for 18 h. Following incubation supernatant collected from the different treatment group and cystine as well as glutamate level measured by UHPLC and GC-MS respectively. For quantification of cystine/glutamate in *ex-vivo* samples, supernatant from the different culture of 4 pups for each treatment groups was pooled together and derivatized thrice independently and run on either UHPLC or GC-MS. Changes in cystine/glutamate were represented as fold change over control.

Analyses were performed using a Nexera X2 Ultra High-Performance Liquid Chromatography (UHPLC) system, equipped with an SIL-30AC autosampler capable of auto-pretreatment function, binary pump system, column thermostat and an RF-20Axs fluorescence detector. Mobile phase (A) constitute 20 mmol/L potassium phosphate buffer (pH 6.5) while (B) was Acetonitrile/Methanol/Water = 45/40/15 (v/v/v). A YMC Triart C18 column with a 0.8 ml/min flow rate maintained at 35 °C in a column thermostat was used to analyze the amino acid content in control as well as treated cell culture supernatant samples. Detection was carried out by an excitation of the OPA-Cystine complex at 350 nm and emission at 420 nm. The eluted peak of Cystine was identified and quantified using an external standard of known concentrations.

Glutamic acid standards prepared with 0.1 N HCl solution were lyophilized before use for obtaining a calibration curve. Similarly, 100 μl of control and treated cell culture supernatent samples were lyophilized. In a typical derivatization protocol, to the above lyophilized sample was added 90 μL of anhydrous pyridine and the resultant mixture was mixed vigorously using cylcomixer till a clear solution is obtained. Analyses were performed using Trace GC gas chromatograph coupled with Quantum XLS mass spectrometer (Thermo Scientific, FL, USA). Helium was used as a carrier gas at a flow rate of 1.1 ml min^−1^. An aliquot of 1 μL of the extracts was injected into the TG-5 MS capillary column (30 m × 0.25 mm i.d. × 0.25 μm film thickness) consisting of a stationary phase of 5% phenyl 95% methyl polysiloxane in the split less mode. Detection was achieved using mass spectrometer in electron impact ionization at 70 eV.

### Measurement of ROS

N9 cells were seeded in a 96 well plate at a density of 10,000 cells/well and grown overnight. 20 min before SA treatment, 50 μM DCFDA dye was added to N9 culture. In different sets, cells were also 1 h pre-treated with NADPH-oxidase inhibitor (100 μM Apocynin and 0.3 μM DPIC) and free radical scavenger N-acetyl cysteine (1 mM) followed by DCFDA and SA (10 μM) treatment. Immediately following SA treatment DCFDA fluorescence intensity was recorded (Ex-485 nm and Em-520 nm) at every 5 min initially followed by every 15 min a microplate reader (BMG labtech) until the level of ROS come down to control level.

### Measurement of cellular GSH level

N9 cells were treated with SA (10 μM) or LPS (1 μg/ml) for 24 h or 4 h either in presence or absence of GSH synthesis inhibitor, BSO. In another set cells were also treated with MRP1inhibitor, cyclosporine A as well as MK571 to find out the role of MRP1 in cellular GSH depletion. To measure cellular GSH level, cells of different treatment groups were harvested and homogenized in 0.1 M potassium phosphate buffer containing EDTA (KPE). Homogenate was centrifuged at 14000 g and supernatant was mixed with 10% trichloroacetic (TCA) followed by vortexing and 10 min incubation on ice. The mixture was centrifuged at 10,000 g for 10 min at 4 °C, supernatant collected and diluted 1:1 with KPE buffer. 10 μl of diluted sample mixed with equal volume of O-phthalaldehyde (1 mg/ml) and 180 μl KPE buffer followed by 10 min incubation in dark. Fluorescence was measured at Ex-355 nm and Em-420 nm in a microplate reader (BMG Omega fluostar). The concentration of cellular GSH was calculated from GSH standard curve^63^.

### Measurement of NO in culture supernatant

NO production by N9 cells in response to SA exposure was measured by a protocol based on Griess reaction^30^. In brief, N9 cells were cultured in phenol red free culture medium in the presence of SA and LPS for 24 h. 50 μl of culture supernatant from control as well as treatment groups were collected and separately treated with equal volume of 1% sulfanilamide solution and incubated for 10 min at room temperature (RT). Following incubation 0.1% NED (1-Napthyl ethylene diamine dihydrochloride) added to it and incubated further for 10 min at RT. The optical density of the solution was recorded in a microplate reader at 540 nm.

### Detection of cytokine level in culture supernatant

Pro-inflammatory cytokine (IL-6 and TNF-α) level in the culture supernatant was measured using Milliplex mouse cytokine assay kit (Millipore) as previously described^4^. In brief, 10,000 N9 cells were seeded in 96 well culture plates and grown overnight. Cells were treated with SA as well as LPS; culture supernatant collected following 24 h incubation and immediately assayed for cytokine through Bio-Plex MAGPIX multiplex reader (Bio-Rad) according to manufacturer instructions.

### Preparation of cell lysate and Western blot analysis

Cells were treated with SA, LPS or other reagents as required for different periods in 6 well culture plates. Following treatment cells were rinsed with cold PBS, scrapped and centrifuged at 4 °C. Cell pellet was resuspended in cell lysis buffer (50 mM Tris–HCl, 150 mM NaCl, 1% TritonX-100, 0.5% sodium deoxycholate, 0.1% sodium dodecyl sulfate (SDS), 2 mM EDTA and 50 mM sodium fluoride) containing 1 mM DTT, 1 mM PMSF and protease inhibitor cocktail. Cells in lysis buffer cocktail freeze-thawed thrice, centrifuged at 12000 g and supernatant were collected. Protein concentration in the supernatant was quantitated by Bradford reagent (Bradford^64^). 50–100 μg of protein of each sample were run on 7–10% SDS-PAGE, transferred to PVDF membrane and probed with primary antibodies of specific interest (1:3000 dilutions in TBS). Desired protein bands were visualized by chemiluminescence substrate in a gel documentation system (G-box H-16, Syngene) and densitometric analysis was done by Image-J software.

### Immunocytochemistry

Immunocytochemistry was performed as previously described^4^. Briefly, cells were grown and treated on glass coverslips in 6 well culture plates. Following treatment cells were fixed in chilled methanol for 30 min at 4 °C and kept in blocking buffer (2% FBS and 0.05% Tween-20 in PBS) 1 h at RT. Cells were incubated overnight with desired primary antibodies diluted in blocking buffer (1:500) at 4 °C. Cells were washed with blocking buffer 3 times for 15 min each and further incubated 2 h at RT with an appropriate secondary antibody. Cells on coverslips were mounted on glass slides with vectashield containing DAPI (Vector lab). Slides were observed under Nikon Eclipse 90i microscope (Nikon, Kawasaki, Japan).

### Immunohistochemistry

Control and SA treated mice were anesthetized and perfused initially with cold PBS followed by 4% paraformaldehyde. The brain was dissected out and kept overnight in the same solution. For cryosectioning brain was washed with PBS and then kept in 15% and 30% sucrose solution for 1 day each respectively. Coronal sections of brain were cut from the posterior side till midbrain using a cryotome (Thermo scientific) and kept in PBS in a 12 well plate. Sections were treated with 0.3% hydrogen peroxide in methanol for 30 min at −20 °C. Brain sections were kept in blocking buffer (2% FBS and 0.1% Triton X-100 in PBS) for 1 h. Following overnight incubation at 4 °C with primary antibody (1:500 in blocking buffer) cells were washed with the same buffer 3 times for 15 min each. Sections were incubated with appropriate secondary antibodies for 2 h at RT. Nuclei were stained with DAPI (1 μg/ml) for 2 min, washed and sections were mounted on glass slides using the antifade mounting medium (Invitrogen). Slides were observed under Nikon Eclipse 90i microscope (Nikon, Kawasaki, Japan).

### TUNEL assay

TUNEL assay was performed by *in-situ* cell death detection kit (Roche) as per manufacturer instructions. Briefly, sections were permeabilized by citrate buffer (10 mM Sodium Citrate, 0.05% Tween 20, pH 6.0) and TUNEL reaction mixture added on the section for 1 h at 37 °C in a humidified chamber followed by washing and mounting on a glass slide. Fluorescent images were captured with Nikon Eclipse 90i microscope (Nikon, Kawasaki, Japan). TUNEL/DCX double positive cells were quantified in 10 μm thick sections in dentate gyrus region of hippocampus from all the respective experimental groups. Data were represented as average TUNEL/DCX double positive cell count per section from four animals in each group^65^.

### Statistical analysis

Results were expressed as mean ± SEM. Statistical significance was determined by unpaired student t-test for two groups and one-way ANOVA for more than two groups, followed by Newman-keuls post hoc analysis by Graph-Pad prism (GraphPad Software, San Diego, CA). p < 0.05 is considered as statistically significant.

## Additional Information

**How to cite this article**: Singh, V. *et al*. Hijacking microglial glutathione by inorganic arsenic impels bystander death of immature neurons through extracellular cystine/glutamate imbalance. *Sci. Rep*. **6**, 30601; doi: 10.1038/srep30601 (2016).

## Supplementary Material

Supplementary Information
