# Supplementary material for: Hijacking microglial glutathione by inorganic arsenic impels bystander death of immature neurons through extracellular cystine/glutamate imbalance
Source: Sci Rep. 2016 Aug 1;6:30601. doi: 10.1038/srep30601 (PMC4967897; doi:10.1038/srep30601)
Supplement: Supplementary Information [file srep30601-s1.doc]

**Supplementary Information**

**Title**

Hijacking microglial glutathione by inorganic arsenic impels bystander death of immature neurons through extracellular cystine/glutamate imbalance

**Name of Authors**

Vikas Singh^*, #^, Ruchi Gera^*,#^ , Rajesh Kushwaha^†, #^ , Anuj Kumar Sharma^*^, Satyakam Patnaik^**^, Debabrata Ghosh^*,‡^

**The affiliation(s) and address (es) of the author(s)**

^*^ Immunotoxicology Laboratory, Food Drug and Chemical Toxicology Group and Nanotherapeutics & Nanomaterial Toxicology Group, CSIR-Indian Institute of Toxicology Research, Lucknow-226001, Uttar Pradesh, India

^#^ Academy of Scientific and Innovative Research (AcSIR), CSIR-IITR Campus, Lucknow-226001, Uttar Pradesh, India

^†^System Toxicology Group, CSIR-Indian Institute of Toxicology Research, Lucknow-226001, Uttar Pradesh, India

^**^Water Analysis Laboratory, Nanotherapeutics and Nanomaterial Toxicology Group, CSIR-Indian Institute of Toxicology Research, Lucknow-226001, Uttar Pradesh, India

**N2a cells exhibit features of immature neuron**

**
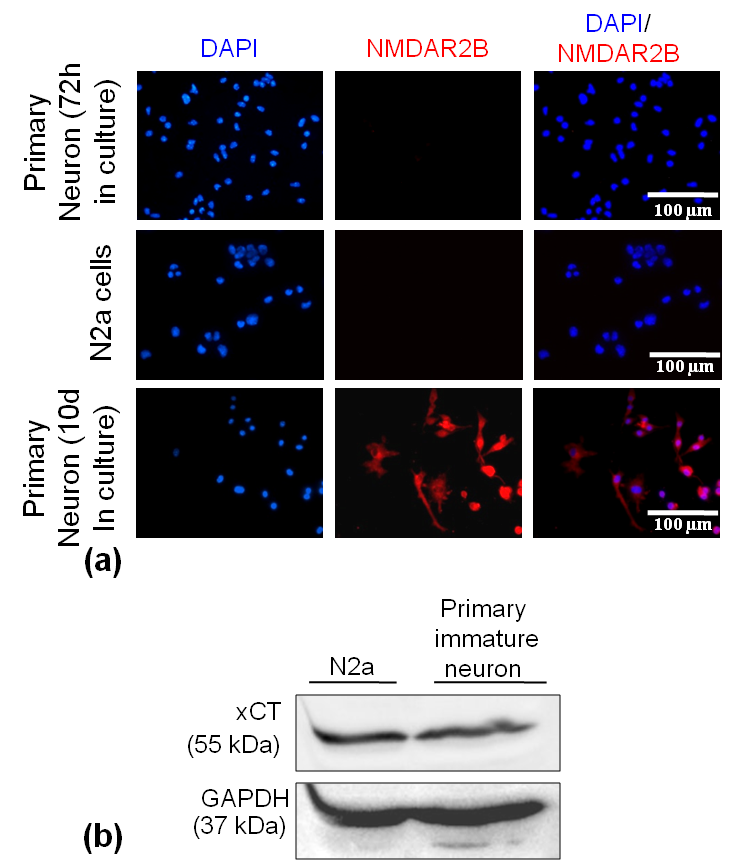
**

**Supplementary Figure S1.** Similarities between N2a and primary immature neuron. (**a**) Primary neurons were isolated from embryonic day 16 pups, cultured for either 72 h or 10 day and immunostained for expression of NMDA receptor 2B (NMDAR2B) which is a marker for mature neurons as well as xCT. Expression of NMDAR2B was neither observed in 72 h old primary neuron culture nor in N2a cells. Whereas primary neurons expressed NMDAR2B following 10 day old culture. (**b**) Western blot analysis revealed that xCT expressed in both N2a and Primary immature neurons.
